# The impact of musical pleasure and musical hedonia on verbal episodic memory

**DOI:** 10.1038/s41598-020-72772-3

**Published:** 2020-09-30

**Authors:** Gemma Cardona, Antoni Rodriguez-Fornells, Harry Nye, Xavier Rifà-Ros, Laura Ferreri

**Affiliations:** 1 Department of Cognition, Development and Educational Psychology, University of Barcelona, 08035 Barcelona, Spain; 2 Cognition and Brain Plasticity Unit, Bellvitge Biomedical Research Institute, L’Hospitalet de Llobregat, 08907 Barcelona, Spain; 3 Institució Catalana de Recerca i Estudis Avançats, 08010 Barcelona, Spain; 4 Laboratoire d’Étude des Mécanismes Cognitifs, Université Lumière Lyon 2, 69676 Lyon, France

**Keywords:** Cognitive neuroscience, Learning and memory, Reward

## Abstract

Music listening is one of the most pleasurable activities in our life. As a rewarding stimulus, pleasant music could induce long-term memory improvements for the items encoded in close temporal proximity. In the present study, we behaviourally investigated (1) whether musical pleasure and musical hedonia enhance verbal episodic memory, and (2) whether such enhancement takes place even when the pleasant stimulus is not present during the encoding. Participants (N = 100) were asked to encode words presented in different auditory contexts (highly and lowly pleasant classical music, and control white noise), played before and during (N = 49), or only before (N = 51) the encoding. The Barcelona Music Reward Questionnaire was used to measure participants’ sensitivity to musical reward. 24 h later, participants’ verbal episodic memory was tested (old/new recognition and remember/know paradigm). Results revealed that participants with a high musical reward sensitivity present an increased recollection performance, especially for words encoded in a highly pleasant musical context. Furthermore, this effect persists even when the auditory stimulus is not concurrently present during the encoding of target items. Taken together, these findings suggest that musical pleasure might constitute a helpful encoding context able to drive memory improvements via reward mechanisms.

## Introduction

Music has a strong emotional power. Accordingly, the most common goal of musical experience is related to music’s ability to modulate emotional state in the listeners^1^. Several studies have shown that, besides recreation, distraction or mood regulation, music-evoked emotions can also drive memory enhancements in both healthy^2–4^ and clinical^5,6^ populations. Most part of these studies focused on two crucial dimensions of musical emotions, namely arousal and valence^7^. In the present study, we aimed at studying the link between music, memory and emotion by focusing on a particular aspect of music-induced affective responses^8^: *pleasure*. Together with motivational and learning aspects, pleasure constitutes a crucial emotional component of reward processing^9^. Pleasure is a complex construct, particularly for humans who, in addition to primary (e.g., food) and secondary reinforcers (i.e., a reward with a learned value associated to a primary reinforcer, such as money), can also trigger rewards from internal mental states (e.g., flow and curiosity^10,11^), intrinsic motivational processes^12,13^, and more abstract rewards such as music and aesthetic appreciation^14^. Music-induced pleasure is strongly related to increases in physiological arousal, such as skin conductance responses (SCR) or heart rate^15^, and to the activity of core reward-related regions within the mesolimbic dopaminergic system^16–21^. Interestingly, humans show music-specific hedonia (i.e., individual differences in sensitivity to musical pleasure, efficiently measured through the Barcelona Music Reward Questionnaire, BMRQ^22^). While listening to music, high-musical hedonic individuals, compared to low- or to music anhedonics, report more intense feelings of pleasure. Furthermore, these subjective ratings are associated to higher SCR as well as higher brain activity and increased functional connectivity in several core regions of the human reward and dopaminergic system^23,24^. Even though music does not provide direct survival advantages, it recruits brain networks similar to the ones activated by primary rewards, such as food or sex. This raises crucial questions about the nature and the possible implications of music hedonic signals.

Crucially, and in line with a wide range of literature showing the importance of emotional significant experiences in memory^25^ and learning^26,27^, recent research has shown that reward is intimately related to memory processing^11,28–30^. At a behavioural level, information associated to potential external rewards (e.g., money or higher point-values in a task where the goal is to earn a larger score)^28,31–33^, internal curiosity states^11^ or intrinsic self-regulated learning processes^34^, lead to better memory performance. According to the neoHebbian framework for episodic memory, this positive memory effect might be related to the interaction between midbrain dopaminergic neurons (substantia nigra/ventral tegmental area complex, SN/VTA) and the hippocampus^35–37^. Reward-motivated activation of midbrain dopamine neurons might indeed result in dopamine release able to strengthen long-term potentiation in the hippocampus, thus enhancing the capacity to store relevant new information into long-term memory^38^ and improving further consolidation processes^39,40^. This would in turn lead to an enhanced recollection associated with better quality, higher confidence and more detailed episodic traces^39,41,42^. Being music a rewarding stimulus triggering dopamine release^21^, it is possible that its positive effect on memory might be at least partially related to the pleasurable responses it triggers^43^. In line with this hypothesis, Ferreri & Rodriguez-Fornells showed that unfamiliar classical music excerpts rated as more pleasant during encoding were significantly better recognized and remembered the next day^44^. Furthermore, they showed that individual differences in the ability to experience reward from music (as measured via the BMRQ^22^) positively predicted memory performance. Notice, however, that this study focused on memory for music itself and it did not explore the possibility of music-related reward responses favouring the consolidation of non-musical material (e.g., verbal information) concurrently present during the encoding process.

The main aim of the present study was, therefore, to determine whether music-driven reward can modulate episodic verbal memory performance for associated items present in the encoding context. To investigate this issue, participants encoded lists of real words in three different auditory contexts: highly pleasant music, lowly pleasant music and white noise. 24 h later their memory was tested with an old/new recognition paradigm. In addition, and in order to test whether the music-related reward responses may specifically influence the recollective episodic experience^39^, we used a remember/know paradigm to differentiate between recollection (‘remember’ responses) and familiarity (‘know’ responses) processes^42^. Furthermore, we employed the BMRQ to evaluate inter-individual differences in musical reward sensitivity. We would expect that words presented in a highly pleasant musical context would be better recognized and remembered than the ones presented in lowly pleasant music or white noise conditions. Moreover, according to our previous results^44^, we would expect different memory performance according to inter-individual differences in the ability to experience musical reward. Specifically, participants with a higher musical hedonia might benefit more from a highly pleasant musical context (i.e., better memory performance) than less musical hedonic participants.

In order to further investigate the link between music reward and memory, the second aim of the present study was to determine whether the positive effect of musical pleasure on memory performance persists even when music is not concurrently present during the encoding of target items. According to the Behavioral Tagging hypothesis (BT^45^), a short-lasting memory induced by a weak event can be consolidated into a long-term memory if paired relatively close in time (usually, within 1 h before until 2 h after the weak event^46^) with an independent and strong event (e.g., a novel or rewarding stimulus). At a cellular level, the weak stimulation sets a tag to a specific synapse where plasticity-related proteins induced by the strong event are captured. This in turn promotes long-term potentiation and creates a persistent mnemonic trace (Synaptic Tagging and Capture model^46,47^). Considering the importance of dopaminergic release in hippocampal long-term potentiation, the memory for events that occur before and after dopamine release would depend not only on their own properties, but also on whether they fell within the penumbra of a dopamine-releasing stimulus^37^. Accordingly, being pleasant music a stimulus triggering dopamine release^21^, it is possible that events occurring not only during, but also after its presentation might result in enhanced memory traces. In order to address this exploratory question, we manipulated the duration of the auditory stimuli during the encoding of verbal material. Half of the participants performed a version of the task in which music was heard before and during the encoding of lists of words (‘with auditory background’ version) and the other half of the sample completed a version in which music was presented only before the encoding (‘without auditory background’ version). According to the penumbra-BT hypothesis, we might expect memory performance enhancement driven by music reward to show no significant differences between these two experimental versions.

## Material and methods

### Participants

**Figure 1:**
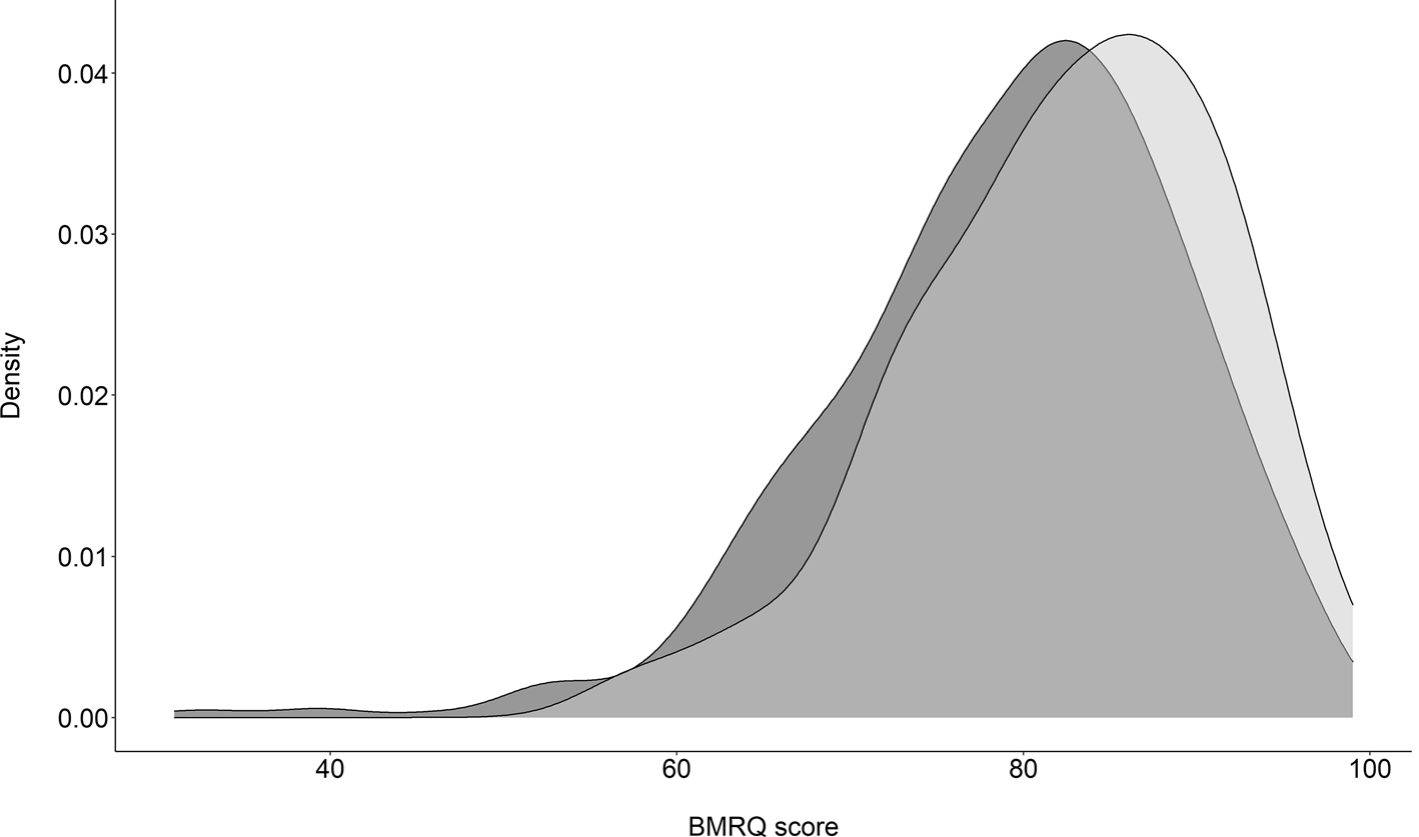
BMRQ score density plots for our sample (light grey, N = 111, 86% female, mean age = 21.1, SD = 2.7) and the sample used in Mas-Herrero et al.^22^ (dark grey, N = 605, 68% female, mean age = 20.5, SD = 3.3).

One hundred and eleven participants (95 female, mean age = 21.1, SD = 2.71), all non-professional musicians (83 non-musicians and 28 amateurs), took part in the study in exchange of university course credits. For each subject, a measure of exposure to classical music (i.e., ‘How often do you listen to classical music?’) was obtained (1–5 scale, from 1: ‘never’ to 5: ‘every time I listen to music, I listen to classical music’). Participants were also asked how much they liked classical music (1–5 scale, from 1: ‘I strongly dislike classical music’ to 5: ‘I love classical music’). All of them were tested with the Physical Anhedonia Scale (PAS^48^) and a modified version of the BMRQ^22^, which included 20 items on musical reward (1–5 scale, from 1: ‘I totally disagree’ to 5: ‘I totally agree’) and two additional items selected to assess amusia (item 21 ‘I can barely perceive when someone is singing out-of-tune’ and item 22 ‘I’m usually unable to recognize a very familiar melody without the help of the lyrics’). The BMRQ shows a 0.92 reliability and it comprises five different facets: Musical Seeking, Emotion Evocation, Mood Regulation, Social Reward, and Sensory-Motor (0.89, 0.88, 0.87, 0.78 and 0.93 reliability, respectively). The BMRQ score density curve in our sample was very similar to the one shown by a demographically comparable sample used for exploratory and confirmatory analyses of the questionnaire^22^ (see Fig. 1).

From the N = 111 sample, two participants reported amusia, five participants resulted general anhedonics and four participants were musical anhedonics. These eleven participants were excluded from the analyses here reported. Thus, the final sample consisted of 100 participants (88 female, mean age = 20.9, SD = 2.57; see Table 1).

**Table 1:** Descriptive statistics (N = 100).

	Age	Classical Music Liking	Classical Music Exposure	PAS score	BMRQ score					
					MS	EE	MR	SM	SR	Overall
M	20.93	3.41	1.95	9.30	14.67	16.75	18	16.12	16.12	83.69
SD	2.57	1.02	1.01	4.30	2.92	2.33	1.69	2.36	2.47	7.57

Classical Music Liking and Exposure range from 1 to 5. For the BMRQ, raw mean additive scores of its five facets [Musical Seeking (MS), Emotion Evocation (EE), Mood Regulation (MR), Sensory-Motor (SM), and Social Reward (SR)] and the overall scale are reported. Maximum score for each facet is 20; with higher scores indicating more sensitivity.

*M* mean, *SD* standard deviation.

The present study was approved by the Ethics Committee of the University of Barcelona. It was conducted in accordance with the Declaration of Helsinki and all participants provided written informed consent.

### Materials

#### Musical stimuli

Musical stimuli consisted of 10 instrumental classical music excerpts. The selection of these excerpts was twofold. First, we selected the 10 excerpts rated as most pleasant and the 10 excerpts rated as most unpleasant from a list elaborated in a previous study^44^. Then, these 20 pieces were additionally rated by an independent sample of 24 participants in terms of familiarity (from 1 = completely unfamiliar to 5 = completely familiar), arousal (from 1 = very relaxing to 5 = very arousing), emotional valence (from 1 = very sad to 5 = very happy) and general pleasantness (from 1 = no pleasantness to 5 = very high pleasantness/chills). From these ratings, we selected the 5 excerpts with the highest (mean = 3.23, SD = 0.14) and the 5 excerpts with the lowest pleasantness ratings (mean = 2.16, SD = 0.36). These two groups (high-pleasure vs. low-pleasure) did not differ in terms of arousal, emotional valence and familiarity (all *p*_*s*_ > 0.05, two-sample *t* tests). Furthermore, we used Spotify’s *Sort Your Music* tool to obtain, for each excerpt, the following attributes: tempo (bpm), energy, valence, and popularity (see Table 2). No significant differences were found between the two groups of stimuli (all *p*_*s*_ > 0.05, two-sample *t* tests). In addition to the musical stimuli, white noise (i.e., a random signal with equal power at any frequency in a given bandwidth) was used as control condition. Each auditory stimulus (i.e., musical excerpts and white noise) was normalized (− 10 dB) and faded (3 s in and 3 s out) with Audacity software (version 2.1.0^49^).

**Table 2:** Musical stimuli.

Title	Artist	Key	T	E	V	P	PR
**Highly pleasant**							
Scherzo capriccioso, Op. 66, B. 131	Antonín Dvořák	B-flat major	122	8	6	1	3.46
Scènes de bal, Op. 17: I. Entrée des masques	Jules Massenet	F major	109	7	25	0	3.25
Suite bergamasque: I. Prélude	Claude Debussy	F major	90	7	11	7	3.21
Symphony No. 8 in F Major, Op. 93: I. Allegro vivace e con brio	Ludwig van Beethoven	F major	146	15	18	0	3.17
6 Pieces for Organ: No. 3 Prélude, Fugue et Variation, Op.18	César Franck	B minor	76	3	17	0	3.08
**Lowly pleasant**							
Concerto grosso in G Minor, Op. 6, No. 6: V. Allegro	George Frideric Handel	G minor	135	26	79	1	2.54
6 Bagatelles, Op. 126: II. Allegro	Ludwig van Beethoven	G minor	71	16	23	2	2.42
3 Intermezzi, Op. 117: III. Andante con moto	Johannes Brahms	C-sharp minor	65	1	8	0	2.29
Mikrokosmos, Sz. 107, BB 105, Vol. 6: No. 149 Dance in Bulgarian Rhythm II	Béla Bartók	C major	101	33	47	2	1.79
Études Symphoniques, Op. 13: Étude VII	Robert Schumann	E major	108	36	9	2	1.75

T = tempo (bpm), E = energy, V = valence, P = popularity. These values were obtained from Spotify’s *Sort your music* tool. PR = mean pleasantness ratings provided by the independent sample.

#### Verbal stimuli

300 Spanish words were selected from EsPal database^50^. These words were 3–10 letter, singular and concrete nouns. The frequency was set at 2–6 per million (database mean = 3.60). Words were divided into 30 blocks with 10 words each one so that there were no significant differences between blocks in terms of number of letters, frequency, concreteness, familiarity and imageability (all *p*_*s*_ > 0.05, repeated-measures ANOVA with a thirty-level within-subjects factor). In addition, semantic relationships between items in each block were avoided to the extent possible. Blocks were divided into two lists, counterbalanced across subjects, one including the items to be encoded and the other one containing the new items randomly mixed with the target items in the recognition test.

### Procedure

**Figure 2:**
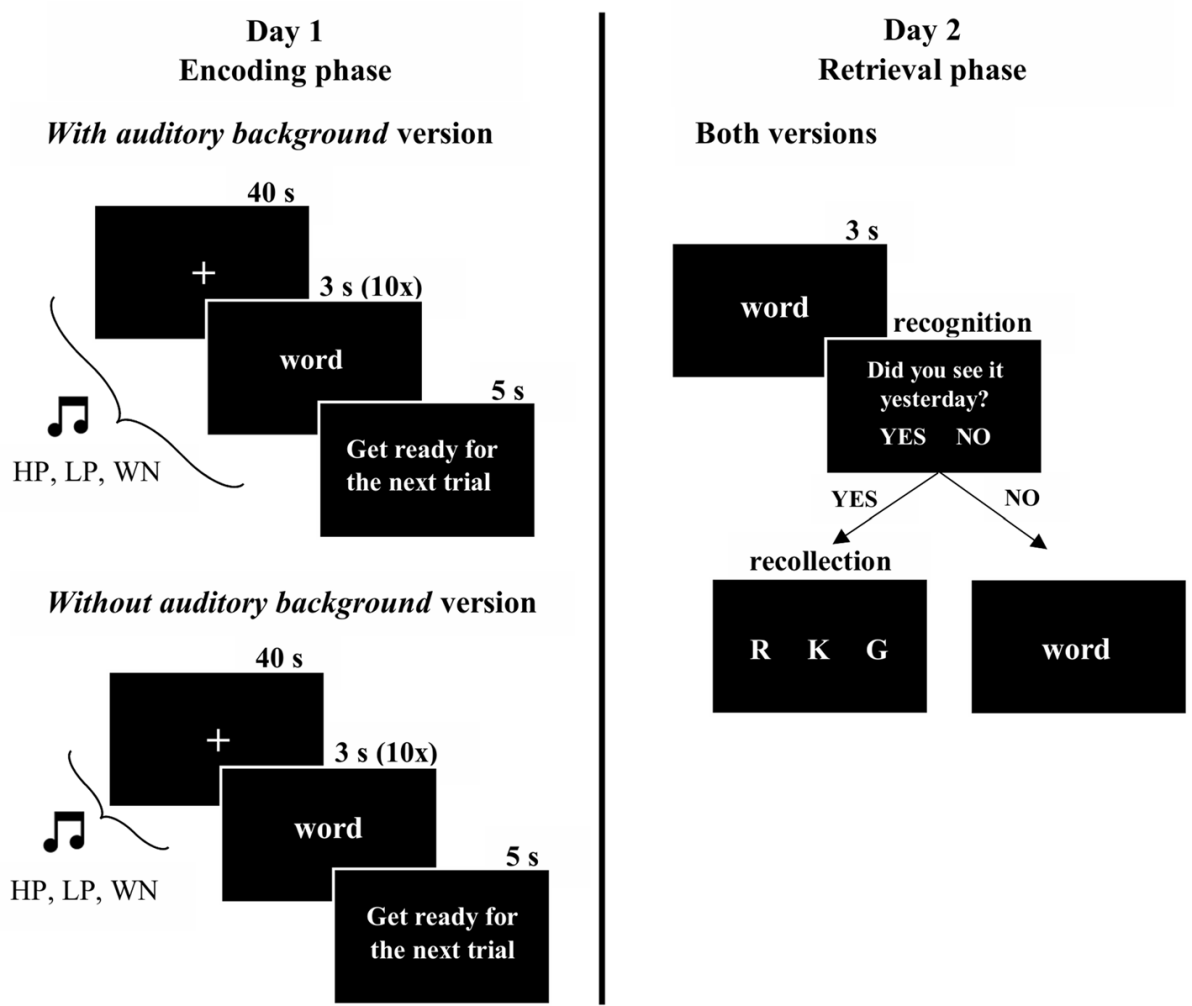
Experimental design. Procedure in day 1 was repeated 15 times corresponding to the 5 excerpts in the three different contexts [Highly pleasant (HP), Lowly pleasant (LP), and White noise (WN)]. Procedure in day 2 was repeated 300 times (150 old words and 150 new words).

On day 1 participants were exposed to 15 encoding blocks (5 highly pleasant music excerpts + 5 lowly pleasant music excerpts + 5 white noise, 10 words for each block). Each block consisted of 40 s of a fixation cross followed by 30 s during which words appeared at the centre of the screen (10 words, one at a time, 3 s each). In the version with auditory background, participants (N = 49) listened to the music/white noise during the whole block (i.e., 70 s, see Fig. 2). In the version without auditory background, participants (N = 51) only listened to music during the first 40 s of each trial (i.e., during the fixation cross, before the encoding) and encoded words in silence (see Fig. 2). Between blocks, participants were allowed to rest for 5 s in silence. Presentation of both lists of words and musical/white noise excerpts were randomized across participants.

24 h later, all participants were presented with 150 old and 150 new words. For each one, they had to indicate if they had seen it the day before (old/new recognition). If so, they had to commit to one of three additional options (recollection task): remember (R), know (K), or guess (G) (see Fig. 2). R indicated that they could recollect something specific about the study episode, K indicated that the word was confidently familiar but they had no recollective experience, and G responses were given when unsure whether the word was old or new (R/K paradigm^42^).

Auditory stimuli were presented using a headset, and the experiment run on E-Prime software (version 2.0; Psychology Software Tools, Sharpsburg, PA, USA).

### Data analysis

We defined as outliers those participants with a d-prime outside the range ± 3 SD. In order to test homogeneity between the groups that completed the different versions (i.e., with or without auditory background) of the task, we ran Student’s *t* tests for those scales following a normal distribution (i.e., BMRQ), Mann Whitney U tests for ordinal measures and for those scales not following a normal distribution (i.e., classical music liking, classical music exposure and PAS) and Chi-Squared tests for nominal measures (i.e., musical expertise). Bonferroni’s correction for multiple comparisons was applied to all statistical significance tests.

In order to test whether different conditions, participants’ musical hedonia and/or the presence or absence of an auditory background during the encoding had a significant effect on recognition performance we used a generalized linear mixed model (GLMM) approach on single trials. The dependent variable (whether the old items were correctly recognized, ‘YES’, or not, ‘NO’) was assumed to have a binomial distribution. Therefore, we applied a logit link function. As explanatory variables we used Condition (i.e., highly pleasant music, lowly pleasant music and white noise) as within-subjects factor and BMRQ score and Background (i.e., with or without auditory background) as between-subjects factors. In order to account for the variability on memory performance from one participant to another, random intercepts for participants were included. BMRQ score predictor was grand-mean centred to avoid convergence failures. Laplace approximation was used for parameter estimation.

Backward elimination method was used for model selection, starting with the full model including all fixed effects (and their interaction) and random intercepts for participants. Increases in model fit were assessed using the likelihood ratio test. Marginal and conditional R^2^ (R^2^_(*m*)_ and R^2^_(*c*)_, respectively) were used as a measure of goodness-of-fit of the final model. R^2^_(*m*)_ describes the proportion of variance explained by the fixed factors whereas R^2^_(*c*)_ explains the proportion of variance explained by both fixed and random factors^51^. Likelihood ratio tests were performed in order to assess the contribution of the different predictors and their interaction to the response variable. Following a significant interaction, pairwise contrasts were used to test how the effect of the continuous variable on memory varied across conditions.

The same analysis was run to test the possible effect of different conditions, participants’ musical hedonia and/or the presence/absence of the auditory stimulus during encoding on recollection performance. In this case, recollection responses were reorganized into two categories (‘Remembered’, including ‘R’ responses, and ‘Not remembered’, including both ‘K’ and ‘G’ responses) and a binomial distribution of the response was assumed.

When using this paradigm, ‘R’ and ‘K’ responses are the most frequently chosen options. Consequently, analyses performed on these responses usually lead to complementary results. However, given that participants can also choose the ‘guess’ (i.e., ‘G’) option, these results could differ. Thus, we performed the analysis for ‘K’ responses. In this case ‘G’ responses were excluded, so the response variable represented whether the recognized word had been further classified as ‘know’ (‘K’ responses) or ‘remember’ (‘R’ responses) and it was assumed to have a binomial distribution.

Analyses were carried out using *lme4*^52^, *emmeans*^53^, and *piecewiseSEM*^54^ packages in R (version 3.6.0^55^).

## Results

Memory performance resulted significantly above chance level for both recognition [*t*(99) = 10.02, *p* < 0.001, *d* = 1.00, one sample *t* test] and recollection [*t*(99) = 5.76, *p* < 0.001, *d* = 0.57, one sample *t* test; see Table 3]. No significant differences in memory performance, musical hedonia, general hedonia, classical music liking, classical music exposure or musical expertise (all *p*_*s *_> 0.121) were found between the two groups that performed the different versions of the task (i.e., with vs without auditory background).

**Table 3:** Memory performance.

	Recognition (hits)			Recollection (‘R’)			Familiarity (‘K’)		
	HP	LP	WN	HP	LP	WN	HP	LP	WN
**With background group**									
M	0.65	0.65	0.64	0.41	0.42	0.45	0.33	0.33	0.32
SD	0.17	0.15	0.17	0.20	0.16	0.18	0.17	0.15	0.14
**Without background group**									
M	0.64	0.65	0.63	0.42	0.40	0.43	0.29	0.30	0.29
SD	0.16	0.15	0.17	0.19	0.17	0.18	0.11	0.12	0.15

Recognition: proportion of old items correctly recognized (i.e., hits; 50 items per condition). Recollection: proportion of hits further classified as ‘remember’. Familiarity: proportion of hits further classified as ‘know’.

*M* mean, *SD *standard deviation.

### Recognition

The full model included Condition, BMRQ score and Background predictors as well as the three double interactions and the triple interaction. Random intercepts for participants were also considered [intra-class correlation coefficient (ICC) = 0.114]. Backward elimination lead to discard all predictors, suggesting that memory performance at the recognition level was not modulated by any of the factors considered in the model.

### Recollection

**Figure 3:**
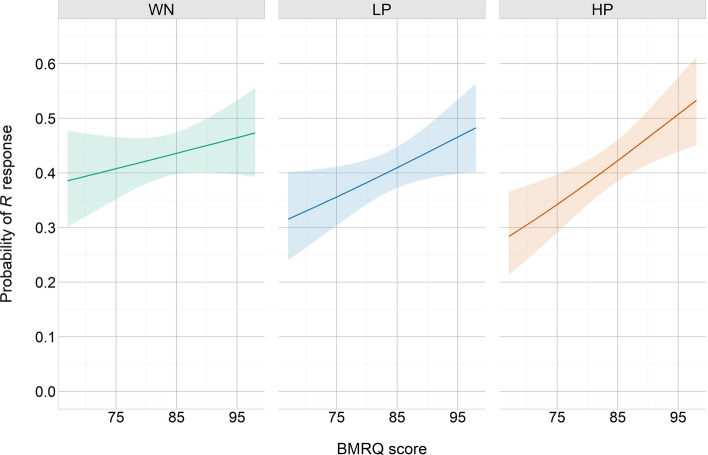
Predicted probability of memory recollection (i.e. ‘R’ response) as a function of participants’ musical hedonia (measured through the BMRQ) in the different conditions [White noise (WN), Lowly pleasant (LP), and Highly pleasant (HP)]. 95% confidence intervals.

**Figure 4:**
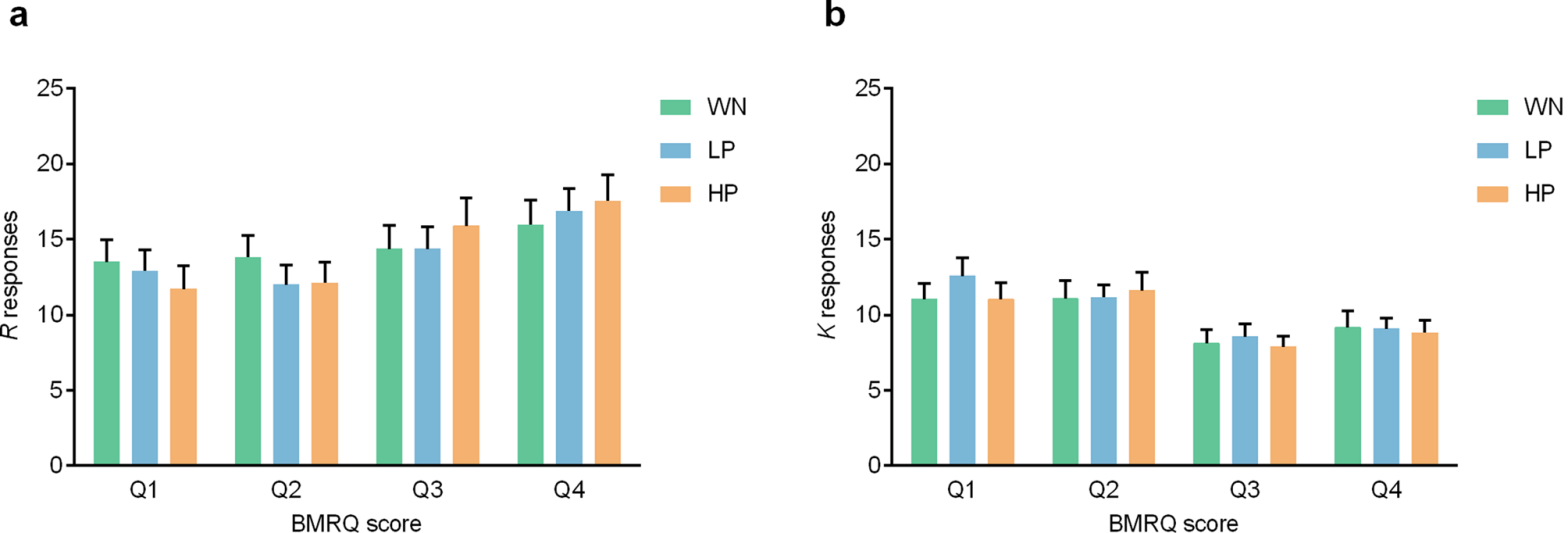
Mean and SEM for total ‘R’ responses (**a**) and ‘K’ responses (**b**) reported in each condition [White noise (WN), Lowly pleasant (LP), and Highly pleasant (HP)] by participants divided into quartiles according to their BMRQ score.

**Figure 5:**
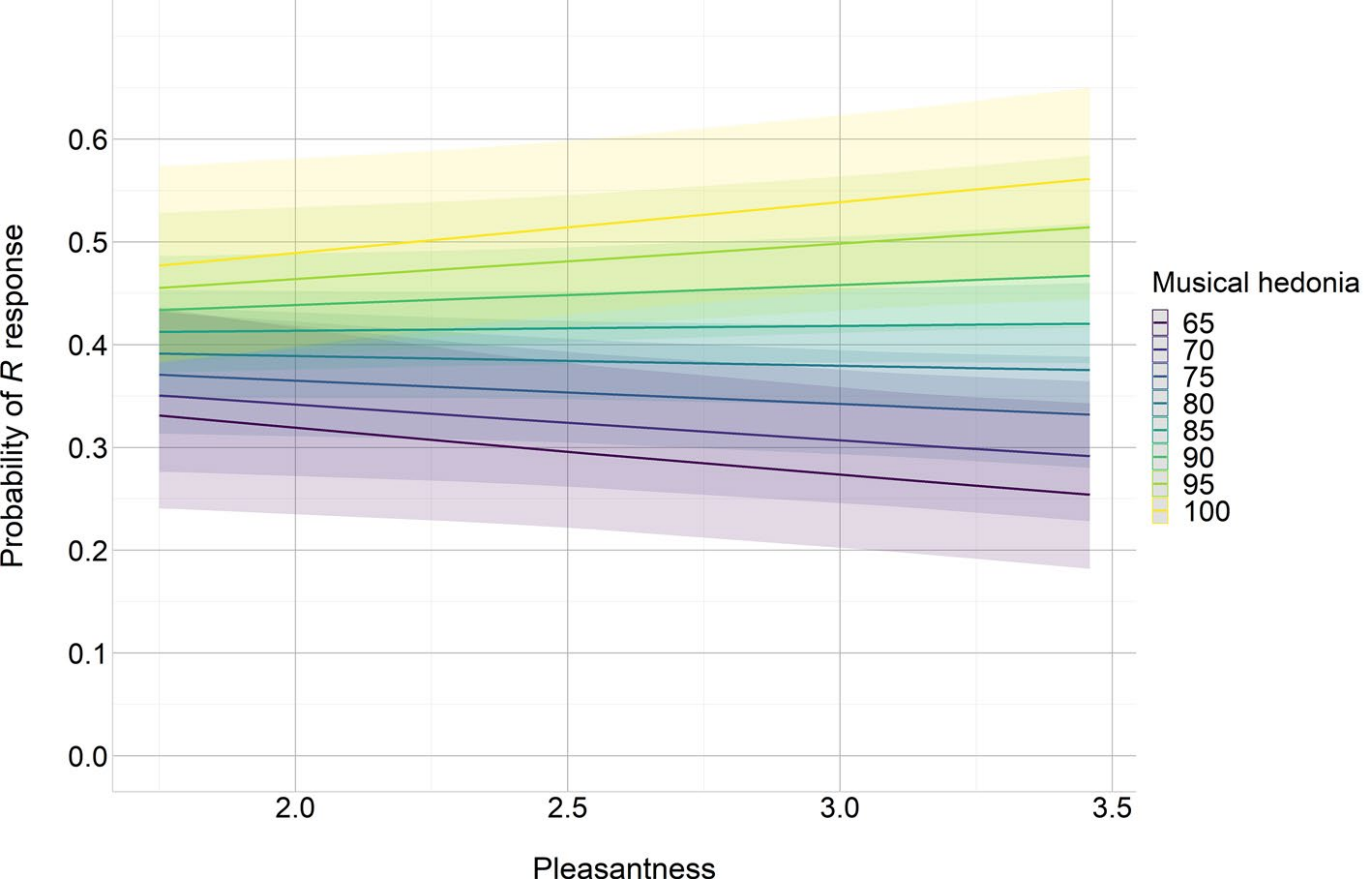
Predicted probability of memory recollection (i.e. ‘R’ response) as a function of musical excerpts’ pleasantness ratings and participants’ musical hedonia (measured through the BMRQ). 95% confidence intervals.

The full model included Condition, BMRQ score and Background predictors as well as the three double interactions and the triple interaction. Random intercepts for participants were also considered (ICC = 0.131). Backward elimination revealed that the best model was the one considering Condition, BMRQ score, and their interaction [${x}^{2}$(5) = 20.9, *p* < 0.001, R^2^_(*m*)_ = 0.008, R^2^_(*c*)_ = 0.110; see Table 4]. Likelihood ratio tests showed a significant effect of BMRQ score [${x}^{2}$(1) = 5.54, *p* = 0.019] and a significant interaction between Condition and BMRQ score [${x}^{2}$(2) = 10.24, *p* = 0.006]. That is, the effect of the different conditions on memory performance was modulated by participants’ sensitivity to music-induced reward (see Figs. 3, 4a). Post-hoc contrasts revealed that the effect of BMRQ score on recollection performance was significantly different between highly pleasant and white noise conditions (*Z ratio* = 3.20, *p* = 0.004). However, no significant differences were found between lowly pleasant and white noise conditions (*Z ratio* = 1.60, *p* = 0.24) or between highly and lowly pleasant conditions (*Z ratio* = 1.63, *p* = 0.23). Given the non-significant differences between the two musical conditions (presumably lead by the small difference in pleasantness ratings between the two sets of musical excerpts used as lowly and highly pleasant; mean rate of 2.16 and 3.23, respectively, on a 5 points scale) we conducted a further analysis considering the mean pleasantness ratings reported by the independent sample for each musical excerpt (i.e., rather than classifying musical stimuli into lowly and highly pleasant categories). Thus, we generated a new model including BMRQ score, Pleasantness and their interaction [${x}^{2}$(3) = 12.5, *p* = 0.006, R^2^_(*m*)_ = 0.011, R^2^_(*c*)_ = 0.109]. Likelihood ratio tests revealed a significant interaction between BMRQ score and Pleasantness [${x}^{2}$(1) = 4.07, *p* = 0.044]. Specifically, for highly hedonic participants, the higher the pleasantness of the excerpt, the greater the probability of remembering the words encoded during/after listening to that excerpt. On the contrary, for less hedonic participants, the higher the excerpt’s pleasantness, the lower the probability of remembering the words associated to that excerpt (see Fig. 5).

**Table 4:** Tested models for recollection.

Fixed effects	df	Measures of fit				Likelihood ratio tests		
		AIC_ci_	∆_*i*_AIC_c_	*w*_*i*_AIC_c_	LL_i_	${x}^{2}$	df	*p*
Cond*BMRQ*Back	13	12,570	9.01	0.01	− 6272			
Cond*BMRQ + Back	8	12,563	1.33	0.32	− 6273	2.34	5	0.801
Cond*BMRQ	7	12,561	0.00	0.63	− 6274	0.67	1	0.412
Cond + BMRQ	5	12,567	6.23	0.03	− 6279	10.24	2	0.006

All models included the Participant variable as random intercept. In the formulas, ‘Cond’ = Condition, ‘BMRQ’ = BMRQ score, ‘Back’ = Background, and ‘*’ = interaction. ‘df’ = degrees of freedom. AIC_ci_ = corrected Akaike Information Criterion. ∆_i_(AIC_c_) = difference between AIC_c_ for model i and best model’s AIC_c_. w_i_(AIC_c_) = Akaike weight measuring the level of support in favour of model i being the most parsimonious among the candidate model set. LL_i_ = natural logarithm of the maximum likelihood for model i. Likelihood ratio tests compare the goodness of fit of that particular model to the previous one. Backward elimination was performed until no further variables or interactions could be removed without a statistically insignificant loss of fit.

In sum, and in line with our hypothesis, episodic memory formation was particularly enhanced when a combination of both highly pleasant musical stimuli and higher sensitivity to experience reward from music were present.

On the other hand, the presence or absence of an auditory background during the encoding did not have a significant effect on recollection performance. Furthermore, the effects of Condition and BMRQ score factors on memory performance were present regardless of the presence or absence of an auditory background, suggesting that music-induced reward favoured the encoding of verbal material not only when it was presented concurrently with the rewarding stimulus, but also when it was presented immediately after.

In light of the essential role of BMRQ score in the predictive capacity of the model, we decided to further investigate this factor. In order to do that, we generated a full model with the five BMRQ facets and their interaction with Condition. Due to the small number of items composing each facet, we used the factor scores estimate instead of the raw addition of items of each subscale^56,57^. Random intercepts were also considered in the model. Backward elimination revealed that the best model was the one including only Emotion Evocation [${x}^{2}$(1) = 9.43, *p* = 0.002, R^2^_(*m*)_ = 0.010, R^2^_(*c*)_ = 0.107], suggesting that the effect of BMRQ score on memory performance was mainly driven by the Emotion Evocation facet.

Finally, analyses for ‘K’ responses revealed that the best model was the one that only included BMRQ score [${x}^{2}$(1) = 10.82, *p* = 0.001, R^2^_(*m*)_ = 0.012, R^2^_(*c*)_ = 0.096]. As expected considering previous analyses, more hedonic participants reported less ‘K’ responses (see Fig. 4b).

## Discussion

In the present study we investigated whether musical reward, and more specifically pleasant musical material and individuals’ musical hedonia, modulate verbal long-term memory. Our results showed that participants with a high musical reward sensitivity presented a greater number of episodic memory traces, especially for words encoded in a highly pleasant musical context. This effect was found regardless of the presence/absence of the auditory stimuli during verbal encoding.

This positive effect of musical pleasantness and musical hedonia on memory is in line with several studies reporting that reward, either extrinsic^28,30,31,33^ or intrinsic^11,34^, can promote memory for relevant and incident information. Nevertheless, these studies used money, point-values, curiosity or self-monitoring of correct performance as reward-eliciting stimuli. One previous experiment focused on the most iconic human abstract reward, music, and showed the effect of music-related reward on musical memory^44^. In the present study, by manipulating a crucial component of reward, pleasantness, we showed a transfer effect of music reward on memory for verbal material. This finding concurs with previous studies highlighting the importance of considering emotional states in predicting memory encoding and consolidation^25,27^ even when the items to be remembered are emotionally neutral^58^.

An important finding of our research is that the pleasant component did not modulate memory performance at the recognition level (i.e., hits), but specifically improved recollection processes (i.e., ‘R’ responses). Traditionally, episodic memory is assumed to rely on two distinct memory processes: recollection and familiarity^42^. Whereas recollection entails the retrieval of specific details associated with the study event, familiarity represents the feeling of having previously encountered the item but not being able to retrieve further details. At a neural level, recollection relies on the hippocampus and prefrontal cortex, whereas familiarity relies on regions surrounding the hippocampus (see^42^ for a review). As the effect of reward on learning and memory is due to an interaction between the reward network and the hippocampus^37^, rewarding stimuli should specifically improve hippocampal-dependent recollection processes. The fact that potential reward responses can specifically stimulate episodic memory performance has been previously suggested by several studies using the dopamine precursor levodopa^39,59,60^. For example, Chowdhury and colleagues^39^ showed that levodopa enhanced memory consolidation and led to a dose-dependent long-term persistent episodic memory improvement (i.e., increase of ‘R’ responses) for images in older adults. Accordingly, our results revealed that more hedonic participants showed an enhanced recollection (i.e., more ‘R’ responses), especially when musical excerpts were highly pleasant, with a subsequent decrease in familiarity (i.e., ‘K’) responses. These results suggest that, for people with a high sensitivity to musical reward, pleasant music constitutes a helpful encoding context that facilitates the retrieval of specific details associated with the study episode. Therefore, it is possible that dopamine-dependent reward responses experienced through pleasant music listening^21^ might facilitate recollective processes for the associated material (i.e., words).

However, although we found that the positive effect of musical hedonia on recollection varied across conditions, post-hoc tests revealed that these differences were mainly driven by the comparison between highly pleasant and control conditions. The non-significant differences between highly and lowly pleasant conditions may be due to the stimuli selection. Indeed, aiming at selecting highly and lowly pleasant stimuli, we avoided the selection of strongly unpleasant stimuli (e.g., dissonant^61^) that could have led to confounding effects on memory (e.g., due to their salience). The two sets of musical excerpts used as lowly and highly pleasant showed a small difference in terms of subjective pleasure ratings (mean rate of 2.16 and 3.23, respectively, on a 5 points scale). This difference, though statistically significant (*p* = 0.001), might have not been enough to induce significantly different states of pleasure leading to significant differences on memory performance. The non-significant differences between lowly and highly pleasant conditions might have been interpreted as a negative result, suggesting that the enhanced memory performance might be due to the presence of music itself (regardless of its pleasantness) or might be the result of a negative affect derived from listening to white noise. However, our further analyses revealed a significant interaction between excerpts’ pleasantness ratings and participants’ BMRQ score, thus confirming that musical pleasantness plays a role in modulating recollection memory performance.

It is noteworthy that music-induced reward responses allowing a better memory performance depend on both: music per se and individuals’ ability to experience reward from music (measured via the BMRQ). Indeed, in line with previous results on musical memory^44^, we showed that only participants reporting higher sensitivity to musical reward benefited from the presence of the highly pleasant musical context. For less hedonic participants, the presence of highly pleasant music during the encoding lead to a negative effect on memory performance. These findings might be interpreted according to a theoretical approach suggesting that musical pleasure relies on the generation of expectations^62^. Midbrain dopamine neurons encode the degree to which an outcome matches expectations: when outcomes are better than expected, a strong activation, known as positive prediction error response, is elicited^63–65^. As these neurons are also active when experiencing moments of intense musical pleasure^17^, it has been proposed that this network might encode reward prediction errors (RPEs) during pleasurable music listening^66,67^. Accordingly, Gold et al.^68^ provided first new evidences that music elicits RPEs in the nucleus accumbens (NAcc). Interestingly, they also found a significant positive relationship between BMRQ scores and RPE signalling in the right NAcc, with less hedonic participants generally reflecting musical RPEs less reliably. Hence, participants with high BMRQ scores might be better at encoding RPEs, which have been recently proposed to play a role in episodic memory formation^69,70^**.** On the contrary, less hedonic participants, although able to predict emotional events as music unfolds, might not experience the expected reward^23,24^. This could lead to a dopaminergic decrease^71^ that would negatively affect encoding processes. Further studies directly addressing the relationship between musical hedonia, RPEs and memory are needed to disentangle such complex interplay. However, it is important to bear in mind that the musical RPE responses based on a cortico-striatal model of musical pleasure might constitute only a part of the global picture. As recently proposed by Goupil & Aucouturier^72^, the pleasurable response to music might be the result of a cooperation between the dopaminergic mesolimbic system and an emotional network involving the amygdala and the dorsolateral prefrontal cortex (DLPFC). Importantly, both the emotional network^7^ and the DLPFC^73–75^ have been shown to be critical in music-dependent encoding and retrieval of information. In line with these findings, the present study revealed that the participants more prone to get emotionally involved with music showed an enhanced memory performance when an auditory context was present during the encoding of information. It might be therefore possible that highest musical reward responses might promote episodic verbal memory formation via a complex interaction of cortical and subcortical networks, in which both pleasure and emotion mechanisms play a crucial role.

Contradictory results have been reported regarding the effect of background music during the encoding on subsequent verbal memory performance^43^. On one hand, some studies suggest that music negatively affects memory performance by attracting participants’ attention away from the to-be-remembered information^76,77^. On the other hand, several studies speak in favour of a general beneficial effect of music on verbal memory^73,74,78–80^. Different mechanisms have been suggested to be responsible for this effect, including the generation of a temporal scaffolding network that supports sequence learning, changes in listeners’ arousal or mood, induction of emotional states, and activation of the reward system^43^. However, so far, no study investigated the effect of experiencing music-related reward during the encoding of non-musical material. The results of the present study suggest that only people with high musical hedonia benefit from the presence of music preceding and accompanying words encoding, especially if the musical stimulus is highly pleasant. Conversely, for less musical hedonic participants, memory performance seems to be better when words are encoded in a white noise context. Therefore, our results suggest that participants’ ability to experience reward from music is a crucial aspect to determine whether music will have a positive effect on their memory.

We found that highly pleasant music and participants’ musical hedonia had an effect on memory performance not only when the encoding of verbal material was accompanied by an auditory background, but also when it was performed in silence immediately after the auditory stimulus presentation. Importantly, this suggests that music-induced reward generates a penumbra that favours the encoding of non-related material presented in close temporal proximity. Most of the studies showing the reward-related penumbra effect so far have used monetary reward anticipation^81^ or a state of high curiosity (i.e., intrinsic reward motivation^11^). In these studies, the encoding of target or incidental items was performed during an anticipatory state, that is, before receiving the previously cued reward. In our study, the to-be-encoded verbal material was presented after the rewarding stimulus. Similarly, a previous study from Fenker and colleagues^82^ investigated the penumbra effect by asking participants to explore novel or familiar scenes before memorizing familiar words. Through a series of experiments, they provided the first evidence that the effect of novelty-induced dopamine release outlasted the triggering event and facilitated the episodic encoding of non-related material up to 30 min later^82^. In the same line, a recent study showed that exposure to an extended block of emotion-evoking stimuli enhanced participants’ recollection of neutral images encoded 9 to 33 min later^26^. These results speak in favour of the possible long-lasting influence of emotion-induced states on prospective memory encoding. Accordingly, our findings suggest that exposure to highly pleasant music, which might be inducing reward responses, favoured the consolidation of information presented immediately after, resulting in an enhanced recollection. However, it is worth noting that the penumbra duration for novel, rewarding and emotional events might be different. As proposed by Wittmann et al.^83^, novelty signals are likely to increase the pool of tonically active dopamine neurons, whereas rewards are more likely to induce phasic bursts of dopaminergic activity. Thus, for example, exposure to novelty could lead to a longer period of elevated tonic firing compared to the one produced by exposure to rewards. In addition, the non-significant differences between with and without background versions reported in the present study and the subsequent interpretation should be taken with caution. Because of the lack of previous experiments studying the penumbra of rewarding stimuli (and particularly music) on memory for weak events, we were not able to perform a power analysis before conducting the present experiment. Therefore, the analyses here reported might be underpowered and, as a result, we might not have been able to detect an effect that was in fact present. Further issues arising from low power and hindering results interpretation include overestimates of effect size and low reproducibility of results^84^. Additional studies are needed to disentangle the duration of the music-driven reward effect on memory.

One possible criticism in the present study concerns the use of white noise as a control condition. Indeed, some authors have reported beneficial effects of white noise on information processing and higher cognitive functions^85–87^. Furthermore, this influence of white noise on cognitive performance has been attributed to dopamine release modulation^88,89^. However, in the learning and memory domain, this positive effect has been mainly observed in children with attention-deficit/hyperactivity disorder (ADHD)^90,91^, while it has been reported from mild to null when tested on healthy people^88,92–94^. Therefore, although results in relation to white noise must be interpreted with caution, the fact that no significant differences in memory performance have been observed between white noise and low-pleasure conditions discards the hypothesis that white noise could have driven a positive effect in this experiment.

Another possible critical point relies on the selection of unfamiliar classical music excerpts. Music-related reward responses can vary depending not only on the inter-individual differences regarding musical hedonia (measured through the BMRQ) but also on musical preferences^15,95^, which can be strongly modulated by previous exposure^67^. Although participants reported a general liking of classical music (M = 3.41, SD = 1.02, on a scale from 1 = completely dislike to 5 = completely like), our results suggest that, if we had selected participants with a specific preference for classical music, the impact of music on memory performance would have been stronger. Furthermore, it is worth to consider that the stimuli were employed as highly or lowly pleasant based on a preselection. In order to not interfere with the memory encoding, no direct reward subjective ratings were recorded during music listening. Therefore, we cannot guarantee that participants were actually experiencing a pleasant stimulus as such. For this reason, although supported by data on individual musical hedonia scores, the interpretation of the results should be done bearing in mind this limitation. An enhancement of reward responses might also have been possible by using participants’ favourite music^21^. However, in that case, we would not have been able to control for other aspects that are likely to modulate music’s effect on memory such as familiarity, emotional valence or arousal.

In conclusion, we have shown that pleasant music contributes to a better encoding of verbal material. We attribute this effect to a greater dopamine release that enhances episodic memory formation thanks to the interaction of the reward networks and the memory circuit. In line with this hypothesis, the positive effect of more pleasant musical context is only observed in those people with the highest ability to experience pleasure from music. Although relevant, these results could constitute only a part of the general picture. In real-life settings, an interplay between changes in arousal and mood, induction of emotional states, and activation of the reward system could jointly contribute to the positive effect of music on memory, and further studies are needed to disentangle such complex interaction.

Taken together, these results might have an important implication for music-based clinical interventions. More specifically, our findings suggest that inter-individual differences in musical reward sensitivity might be a critical predictor of the effectiveness of a music-based treatment for memory stimulation (see also^96^).

The present study showed that musical pleasantness, able to modulate music-related reward responses, and musical hedonia play a crucial role in driving the positive effect of music on verbal episodic memory. In our opinion, this sheds new light on the relationship between music, reward, and memory, thus opening important perspectives about the use of music for the stimulation and rehabilitation of memory processes.

## Data Availability

The datasets generated during the current study are available in the OSF repository (link: https://osf.io/krz5p/?view_only=da31a5077dee4c97bf3ab40cb1e21fd6).
